# Supplementary material for: Are carbon emissions trading and green financial instruments synergistic? -Comprehensive quantitative research based on content analysis
Source: PLoS One. 2024 Mar 7;19(3):e0298601. doi: 10.1371/journal.pone.0298601 (PMC10919601; doi:10.1371/journal.pone.0298601)
Supplement: S1 Data — (ZIP) [file pone.0298601.s001.zip › Data/地级市Z_F工作报告.zip!/║■▒▒/╩«╤▀╩╨/└┤╫╘╣1⁄2╓┌║┼í╛┬φ┐╦╩2╛▌═°í┐_2005.docx]

各位代表：

　　本届政府从2000年任职，迄今五年届满。现在，我代表市人民政府，向市第三届人民代表大会第一次会议报告过去五年的工作，对今后五年的政府工作提出建议，请予审议，并请各位政协委员和列席会议的同志提出意见。

　　一、过去五年政府工作的回顾

　　过去五年，是我市全力推进改革开放，国企改制、招商引资取得重大突破的五年；是着力调整经济结构，特色经济、县域经济发展强劲的五年；是全面加强基础设施建设，交通、水电和城镇建设成效显著的五年。五年来，在市委的正确领导下，全市上下坚持以邓小平理论和“三个代表”重要思想为指导，紧扣发展主题，抢抓历史机遇，沉着应对各种困难，努力化解不利因素，国民经济快速发展，社会事业全面进步，政治文明和精神文明建设迈出了新的步伐。

　　（一）国民经济快速发展。

　　五年来，不仅顺利实现了“九五”计划，而且提前一年超额完成了“十五”计划的主要任务。市二届人大一次会议确定的本届政府五年任期目标全部实现，其中生产总值、财政收入、固定资产投资等指标大幅度突破。全市生产总值由1999年的167.47亿元增加到2004年的290.9亿元，超计划目标24.3%，五年年均递增10.6%，人均生产总值首次突破1000美元大关；财政收入超计划目标37.6%；全社会固定资产投资超计划目标72.2%。2002年和2003年我市两度获得“全国综合实力百强城市”殊荣。

　　农村经济全面发展。高度重视“三农”工作，认真落实国家惠农政策，坚持调整农业结构，特色经济和县域经济发展步伐加快。2004年五县一市实现生产总值109.3亿元，年均递增7.8%。农业结构调整显现成效。特色产业基地发展到300万亩，特色产业产值占农业总产值比重提高到80%以上。农民生产生活条件得到改善。除险加固病险水库70座。建成各类饮水工程4.4万处，解决了40万人饮水困难，我市被国家水利部授予“农村饮水解困先进集体”。完成城乡电网改造任务，实现了城乡同网同价。修筑通乡油路912公里、通村公路794公里。新阶段扶贫开发工作初见成效。五年来，争取扶贫资金12.2亿元，实施扶贫项目1500余个，首批省定63个重点贫困村具备申请验收条件，特困人口、低收入人口分别减少15.1万人和10.1万人。

　　工业经济实力进一步增强。坚持服务东风公司不动摇，巩固壮大汽车主导产业；充分利用优势资源，大力发展新兴支柱产业，工业经济速度和效益同步增长。2004年工业总产值突破500亿元大关，达到513.2亿元，年均递增14.2%；规模以上工业企业实现增加值134.8亿元，年均递增14.9%；工业经济效益综合指数达142，比1999年提高67个点。汽车、医药、电力工业产值分别年均递增14.4%、42.5%、26.3%，化工、冶金、轻工、纺织、建筑建材等产业规模也不断壮大。

　　第三产业发展水平明显提升。2004年第三产业实现增加值94.9亿元，年均递增10.5%。旅游产业长足发展。2004年全市接待国内外游客540.3万人次，实现旅游总收入21.9亿元，创汇670.1万美元，年均分别递增12.9%、15.2%和19.8%，我市荣获中国优秀旅游城市称号，武当山被评为全国首批4A级旅游区。商贸流通日趋活跃。2004年实现社会消费品零售总额105.7亿元，年均递增10%；中国（十堰）汽配城年销售额超过40亿元，汽配广场物流中心建成运营。信息产业迅猛发展。2004年完成邮电业务总量7.3亿元，年均递增14.6%；宽带城域网基本建成；城乡固定电话用户达到38万户，比1999年增长1.5倍；移动电话用户达到39万户，比1999年增长9.5倍。房地产业发展迅速。累计完成房地产开发投资36.5亿元，年均递增36.4%；启动了住房二级市场，房地产交易额15亿元；初步建立了住房公积金制度，发放个人公积金住房贷款1.3亿元。

　　财政金融运行平稳。克服政策性、体制性减收因素多的困难，努力增收节支，财政收入持续增长。财政收入由1999年的13.6亿元增加到2004年的27.52亿元，年均递增15.1%，其中地方一般预算收入由7.39亿元增加到10.16亿元，年均可比增长12%。实现了财政收支的分年分级平衡，基本保证了县市工资按时发放。强化金融监管，防范和化解金融风险，区域金融保持平稳。2004年末，全市金融机构各项存款余额357.7亿元，比1999年增加188.1亿元；信贷投入总量355.3亿元，比1999年增加116.6亿元；不良贷款由1999年的92.5亿元减少到2004年的44.7亿元。

　　（二）项目建设成效显著。

　　坚持以项目为载体，积极争取和吸纳国家建设资金及境内外资金，加快基础设施和生产性项目建设步伐。全社会固定资产投资由1999年的30.19亿元增加到2004年的77.5亿元，年均递增20.8%。

　　交通建设突飞猛进。银沪高速公路许土段竣工通车、十漫段开工建设，结束了十堰境内无高速公路的历史。郧庙路、十巫路、316国道十白段和鲍竹路改造竣工通车，丹江口二桥、将军河大桥建设进展顺利，改造干线公路1048公里、等级公路1274公里。目前，城区至县市、各县市之间及主要省级出口路已全部达到山区二级路标准，交通瓶颈制约得到缓解。

　　城镇基础设施不断完善。综合改造了十堰、武当山火车站，改造了人民路、公园路和东风公司移交的道路等23条，新建了北京路、东风大道，建设了神定河污水处理厂和刘家沟、丁家湾垃圾处理场；完成了市热电厂“四炉两机”工程，城区居民用暖状况得到改善。全市城镇化率达到34%，比1999年提高6.6个百分点。运用经营城市理念，实现社会直接融资21亿元，加快了城镇建设步伐。

　　生产性项目建设顺利推进。水电建设取得历史性突破。陡岭子、孙家滩等16处电站竣工投产，黄龙滩扩建工程首台机组投产发电，新增装机容量29万千瓦；黄龙滩扩机、鄂坪、松树岭、三里坪等27处工程正在抓紧建设，装机容量50万千瓦；潘口、龙背湾、孤山等水电站前期工作进展顺利，装机容量突破100万千瓦。电网建设步伐加快。丹江口、柏林、竹山220千伏输变电工程开工建设。技改项目进展顺利。全市累计完成技改投资72.9亿元，年均递增19.3%。丹江电解铝、农夫山泉一期、清大药业、轿车车身开发等重大技改项目相继建成投产。秦岭山区农业综合开发、农村能源建设等项目有序推进。服务南水北调工作全面展开。经过争取，征地移民安置补偿补助标准大幅提高，中线水源有限公司落户丹江口市，一批库区重点项目列入相关规划，南水北调中线工程加坝施工大桥开工建设。

　　（三）改革开放取得新突破。

　　国企改革深入推进。坚持以民营化为取向，全面推进国有集体企业改制，第二轮952家企业改制任务基本完成。积极推进以市公交公司改革为重点的市政公用行业合资改制工作，我市率先在全国实现了城市公交整体民营化。市车架厂成功拍卖，市建筑总公司与湖北卓越集团顺利重组，改制重组后的企业实力进一步增强。全力支持中央、省驻市企业分离办社会职能，先后接收城区部分道路和中小学、公安、消防、医院等机构。

　　农村改革不断深化。进一步深化农村税费改革，全面实行涉农收费公示制和农村中小学“一费制”，取消不合理收费项目331项，降低收费标准206个，全市农民人均负担由改革前的111.4元减少到目前的27.6元，下降75.2%。加快推进乡镇综合配套改革，全市乡镇（街办）减少30%，事业单位减少67%，财政供养人员定编精减40%。

　　财政体制改革顺利实施。认真落实省管县（市）财政体制改革的政策，新旧体制衔接有序。切实加强预算管理改革，部门预算自上而下展开，市直一级单位部门预算覆盖面达100%。推进国库集中收付制度改革，市直11个部门纳入了改革试点。规范收支两条线管理，预算外资金专储率和收支计划管理面分别达到98%、99%。政府采购范围和规模不断扩大。职务消费货币化改革逐步推进。

[FS:PAGE]

　　对外开放水平不断提高。招商引资工作取得新进展。五年来，全市新批外商投资企业92家，注册资本3.1亿美元。实际利用外资由1999年的742万美元增加到2004年的3750万美元，年均递增38.3%；利用市外境内资金由1.3亿元增加到24亿元，年均递增79.6%。开发区成为对外开放的重要窗口。十堰高新区累计引进500万元以上的项目112个，引进资金12.5亿元。外贸出口呈现多元化发展格局。五年共有68家企业获得自营进出口权。全市实现外贸出口由1999年的2623万美元增加到2004年的4507万美元，年均递增11.4%。十堰海关获准设立，海关大楼即将建成运营。

　　（四）社会事业全面进步。

　　科技、教育事业快速发展。进一步加大科技投入，推进科技体制创新，科技进步对经济增长的贡献率达到46%。五年来，开发高新技术产品143个，专利申请量1172项，获省级科技奖励31项，地膜水稻和鄂玉10号、16号新品种等农业科技创新成果得到广泛推广，“常差速常互锁传动装置技术”获省技术发明一等奖，并获得19个国家和地区专利。我市被科技部批准为全国农村信息化科技试点市，十堰高新区被批准为国家火炬计划汽车关键零部件产业基地，张湾、茅箭被评为全国科技先进区。认真落实基础教育以县为主的管理体制，推行全员聘用制，调整农村中小学布局，加强学校卫生安全管理，教育规模不断扩大，教育质量不断提高。2004年小学适龄儿童入学率达到100%，初中阶段在校学生入学率96%，高考上省线率高于全省平均水平，高等教育和职业教育继续发展。建立人才引进“绿色通道”，累计引进各类急需人才700多名，为经济社会发展提供了智力支撑。

　　文化体育、广播电视、医疗卫生事业健康发展。广泛开展群众文化活动，成功举办“武当山”、“南水北调”特种邮票首发式和武当国际旅游节，首次启动了“9·15”武当山世界文化遗产保护日活动。大力开展全民健身活动，市体育中心建成投入使用，成功承办省第十一届运动会。全面发展广播电视事业。建成广电光纤网3000多公里，有线和无线综合覆盖率达到95%，基本实现了村村通广播电视。加快发展卫生事业。出台了《十堰市公共卫生建设三年规划》，重点加强紧急救援中心、市县疾控中心、传染病院和乡镇卫生院发热门诊建设，公共卫生基础条件得到改善；大力发展社区卫生服务业，积极推行新型农村合作医疗试点，城乡就医环境进一步优化。非典和禽流感防治取得胜利，艾滋病、结核病等传染性疾病得到有效控制。

　　就业和社会保障工作取得新成效。认真落实就业再就业政策，广辟就业渠道，五年累计安置新成长劳动力和下岗失业人员就业再就业15万人，城镇登记失业率控制在4.2%以内。加强社会保障体系建设，认真做好“三条保障线”的衔接工作，确保了国有企业下岗职工基本生活费和企业离退休人员养老金、失业救济金100%发放；困难职工援助中心发挥了积极作用，各级劳模得到较好照顾；建立了最低生活保障制度，7.8万城镇贫困居民纳入低保范围，实现了应保尽保；实行农村特困户定期定量救助制度，2.8万人纳入农村贫困救助范围；稳步推进“福星工程”，五保对象集中供养达70%。扎实开展抗灾救灾工作，战胜了汉江20年来最大的洪水和局部暴雨、山洪、山体滑坡等多种自然灾害，妥善安置了灾区群众的生产生活。

　　人口资源环境协调发展。高度重视人口与计划生育工作。人口自然增长率降至4‰以下，符合政策生育率提高到98%。加强耕地保护。开发整理新增耕地5.2万亩，连续五年实现全市耕地总量的动态平衡。实施生态立市战略。退耕还林、天然林保护等国家生态保护与建设工程顺利推进，完成退耕还林173万亩，管护森林资源1059万亩，治理小流域105条。我市通过了全国生态示范区验收，并再次荣获“国家园林城市”殊荣。

　　（五）政治文明和精神文明建设进一步加强。

　　民主建设继续推进。各级政府坚持定期向人大报告工作，向政协通报情况，重大决策征求人大、政协、民主党派、党外人士和人民团体意见，自觉接受法律监督、民主监督。五年来，市政府累计办理人大代表议案、意见、建议881件，办理政协委员提案1498件，办结答复率100%。高度重视基层民主建设，顺利完成了县市区换届选举和第五次村委会直选。

　　依法行政水平不断提高。认真贯彻实施《行政许可法》，成立了政府法律咨询委员会，全面清理了行政规范性文件，加大了行政执法监督力度。深化行政审批制度改革，先后取消、调整行政许可和审批事项627项，市级许可和审批事项减少47.7%。深入开展普法教育，完成了“三五”普法的检查验收，启动实施了“四五”普法工作。进一步提高政务工作透明度，重新修订了《政府工作规则》，完善了相关制度和工作程序；成立“行政服务中心”，办结了10.2万件审批服务事项。改进政府作风，加强“市长公开电话”工作，坚持市领导接待群众来访制度，成立了“经济发展环境投诉中心”，开展了民主评议行风、企业评议政府部门、社会公众评议政府机关等活动。加强廉政建设，加大行政效能监察和审计监督力度，我市被授予“全国经济责任审计工作先进地区”光荣称号。

　　社会保持稳定。加大社会治安综合治理力度，依法打击各类违法犯罪活动，坚持不懈地同“法轮功”等邪教组织作斗争，认真做好“法轮功”人员的教育转化工作，我市被省委省政府授予“全省社会治安综合治理优胜单位”称号，市公安局被评为“全国严打整治斗争先进集体”。积极排查和化解矛盾，妥善处置群体性事件260多起。强化安全生产监管，建立重特大事故防范预警机制和监督体系，开展专项整治活动，完成城区煤气管网、百二河河道市场等重大安全隐患整改工作，有效遏制了重特大事故的发生。强化食品、药品质量监管，严厉查处药品违法经营和制售假劣药品、医疗器械违法行为，实行猪牛羊定点屠宰，有效保证了人民群众饮食和用药安全。

　　精神文明建设向纵深推进。以创建国家级文明城市为动力，积极开展文明社区、文明单位、文明农户等系列创建活动，城乡精神文明建设整体水平不断提高。采取多种形式开展形象宣传活动，进一步提高了城市知名度。全市档案系统获“全国先进集体”称号，市交通局、市审计局被评为全国精神文明建设先进单位，太和医院被评为全国文明行业先进单位。强化全民国防教育，深入开展“双拥”活动，全面落实拥军优抚政策，积极支持部队和国防建设，进一步加强民兵预备役建设，我市实现了国家和省“双拥模范城”四连冠，并获得全国“人民防空先进城市”称号。

　　（六）人民生活水平不断提高。

　　2004年城镇居民人均可支配收入6680元，年均增加454元；农民人均纯收入1916元，年均增加97元。居民人均储蓄存款6141元，比1999年增加3179元。居民人均消费水平由1999年的2326元增长到3686元。城乡人均居住面积分别由13.9平方米、26.1平方米提高到18.5平方米和28.1平方米。各类饮用水源水质达到二类标准，空气质量保持在一级以上。

　　在三个文明建设中，外事、侨务、统计、机关事务管理、民族宗教、地震、气象、水文、对台、社科、老龄、残联、方志等方面工作都取得了新的成绩。

　　刚刚过去的2004年，全市上下坚持科学发展观，着力改善发展环境，加大改革开放力度，加快经济结构调整，狠抓重大项目建设，全市经济整体呈现积极向上的好形势，各项社会事业都有新的发展，国民经济各项指标均全面完成或超额完成年初计划。全市生产总值同比增长16.3%，财政一般预算收入可比增长29.9%，全社会固定资产投资增长32.1%，社会消费品零售总额增长13.4%，外贸出口增长27%，实际利用外资增长22.9%，农民人均纯收入增长6%，城镇居民人均可支配收入增长7.4%，人口自然增长率和城镇登记失业率控制在计划目标以内。

[FS:PAGE]

　　各位代表，上述成绩的取得，是全市人民团结奋斗、辛勤劳动的结果，是市委正确领导的结果，是市人大、市政协监督支持的结果。在此，我代表本届政府，向所有参与、支持、关心十堰发展的同志们、朋友们表示衷心的感谢和崇高的敬意!

　　在总结成绩的同时，我们也清醒地认识到，我市经济社会发展中仍存在一些突出矛盾和问题：城乡之间和不同阶层之间的收入水平差距明显，并呈拉大的趋势；农业基础设施落后，抗灾保收能力较低，粮食增产、农民增收的基础仍不稳固；经济结构单一，经济外向度低，经济整体竞争力不强；就业再就业压力大，社会保障体系不够健全，部分下岗职工、城镇低收入居民和农村贫困群众的生活还比较困难；政府在转变职能、改进作风、优化环境等方面还存在很多不足。这些矛盾和问题，需要在今后工作中认真加以解决。

　　二、今后五年政府工作的基本思路和主要目标

　　今后五年，是我市发展史上的重要战略机遇期。国家实施中部崛起战略，推进新阶段扶贫开发，支持贫困地区和革命老区建设，将进一步促进十堰城乡协调发展；南水北调中线工程全面开工建设，丹江大坝加高、水源区生态保护和库区移民等重大项目的实施，十堰将成为国内外广泛关注的调水源头；东风汽车有限公司全面实施中期事业计划，加大商用车重大战略项目的投资力度，“中国第一、世界前三”目标的实现，十堰将成为名符其实的国际化商用车产业基地；银沪高速公路十漫段和襄渝铁路复线的建设，高速公路和快速铁路双双贯通，十堰将成为连接我国东西部的交通大通道；汉江——堵河流域水能资源的集中开发，电站和电网建设步伐加快，十堰将成为湖北重要的水电基地。这些不可多得的重大机遇，我们要倍加珍惜，进一步坚定发展信心，加快发展步伐。

　　今后五年政府工作的基本思路是：以邓小平理论和“三个代表”重要思想为指导，认真贯彻党的十六大精神，抢抓历史机遇，全面落实科学发展观，坚持大开放工作思路，继续实施工业兴市、生态立市、人才强市战略，全面深化改革，着力调整经济结构，转变经济增长方式，大力发展“一主四大”产业，全力服务东风公司和国家重点工程建设，加快国际化汽车城、水电城、旅游城、生态城和区域性中心城市建设步伐，努力构建和谐十堰，促进经济社会全面协调可持续发展。

　　建议未来五年经济社会发展的指导目标是：生产总值年均增长9%左右，财政一般预算收入年均增长10%左右，全社会固定资产投资年均增长12%左右，社会消费品零售总额年均增长9%左右，利用外资年均增长10%左右，外贸出口年均增长10%左右，城镇居民人均可支配收入年均增长6%左右，农民人均纯收入年均增长5%左右，城镇登记失业率控制在5%以内，人口自然增长率控制在6‰以内。全面完成上述目标任务，我市经济实力将跃上一个新台阶，社会发展将实现历史性跨越，人民群众生活质量将有较大幅度提高。

　　为实现上述目标，在今后的工作中，要坚持以下几个原则：一是可持续发展原则。以科学发展观统领全局，并切实贯穿于全市经济社会发展的各个方面，实现经济、社会、资源和环境协调发展，三个文明齐头并进。二是突出重点原则。在统筹兼顾的同时，集中力量抓重点、办大事、打基础，突出抓好经济结构调整、城乡居民增收、交通设施建设、就业和社会保障等事关全局的关键性工作。三是改革创新原则。全面深化改革，推进体制创新、技术创新、管理创新，不断增强经济社会发展的体制活力和内生动力。同时，继续实施大开放战略，努力提高对外开放水平，更好地利用国内外两个市场、两种资源，加快外向型经济的发展。四是依法行政原则。严格依法办事，积极履行职责，提高行政效能，努力形成行为规范、运转协调、公正透明、廉洁高效的行政管理体制，创造良好的发展环境，为市场主体提供优质服务。五是执政为民原则。坚持以人为本，把维护与实现广大人民群众的利益作为一切工作的出发点和落脚点，着力解决关系群众切身利益的突出问题，不断提高人民群众的生活水平和质量。

　　三、2005年政府工作的建议

　　2005年是新一届政府施政的开局之年，是“十五”计划的最后一年，做好今年的工作，具有承前启后的重要意义。今年全市经济社会发展的主要预期目标建议为：生产总值增长10%左右，全社会固定资产投资增长16%，财政一般预算收入可比增长12%，社会消费品零售总额增长10%，利用外资增长15%，外贸出口总额增长15%，城镇居民人均可支配收入增长6%，农民人均纯收入增长5%，居民消费价格涨幅低于4%，城镇登记失业率控制在4.5%以内，人口自然增长率控制在省定目标以内。经过努力，今年我市经济可望实现五个突破：生产总值突破300亿元，固定资产投资突破100亿元，财政收入突破30亿元，农民人均纯收入突破2000元，城区居民人均可支配收入突破1万元。

　　（一）全面发展农村经济，千方百计增加农民收入。

　　提高粮食综合生产能力。实行最严格的耕地保护政策，加强基本农田保护。进一步落实中央支持粮食生产的政策，加大对粮食生产的补贴和投入。组织实施“农业科技落实年”活动，积极推广应用农业先进适用技术。继续实行“以膜代补、市县配套”的扶持政策，推广地膜玉米20万亩、水稻6万亩、油菜3万亩。力争粮食总产15亿斤以上。

　　大力发展特色经济。坚持区域化布局、规模化发展，高标准新建茶叶、柑桔等八大特色产业基地30万亩。抓好高速公路沿线地区产业结构调整，培植特色农产品生产和加工产业带。加快培育和引进龙头企业，引领农业产业化开发。大力发展无公害、绿色、有机食品，建立农产品加工业的技术创新体系，着力开发具有地方特色的农产品品牌，提高特色经济的整体竞争力。

　　加快发展畜牧业。通过小额贷款等多种方式加大投入，大力发展山羊、优质猪生产，扩大肉牛和土鸡规模养殖，积极发展奶牛产业。依靠科技，做好畜禽品种改良和疫病防治工作。抓好畜禽产品的深加工，提高产品附加值。力争畜牧业产值增长15%以上，畜牧业收入在农民收入中的比重明显提高。

　　进一步发展打工经济。组织实施好农村劳动力转移培训“阳光工程”和科技扶贫培训项目，提高农民综合素质。继续完善措施，加大就业指导、信息服务、社会保障等方面的工作力度，提高劳务输出的指导效率和组织化程度。大力发展能人经济，鼓励能人组织农村剩余劳动力外出务工，支持能人回乡投资兴业。加强对劳务市场的监督和跟踪管理，解决劳动环境差、拖欠农民工工资、侵害农民工权益等突出问题，切实保障务工农民的合法权益。

　　扎实推进扶贫开发。切实加强重点贫困村建设，突出整村推进、产业化扶贫和促进农村剩余劳动力转移三个重点，坚持解决温饱和巩固温饱同时推进，全面完成283个重点贫困村建设任务，启动省新定107个重点贫困村建设项目。努力增加贫困人口收入，力争全市贫困人口减少9万人。加大对城区贫困村扶持力度，重点解决行路难、通讯难问题。强化扶贫项目资金管理，严格执行扶贫项目资金“公示制、预拨制、报账制”管理办法和项目资金审计制度，提高扶贫资金的使用效益。

　　加强农村基础设施建设。突出抓好农田水利、节水灌溉等基础设施建设。改造基本农田4.8万亩，除险加固水库42座，维修塘堰2000口，治理水土流失面积180平方公里。大力推进农村饮水解困、饮水安全、沼气池等工程建设。

　　（二）大力发展工业经济，推进新型工业化进程。

　　发展壮大汽车主导产业。坚定不移地做好政企共建工作，全力支持东风汽车有限公司实施中期事业计划，谋划建设东风汽车工业园，为东风公司发展营造良好环境。大力支持东风实业、东风轮胎、三环专汽等重点企业发展。引导中小企业加强经营管理，提升技术层次，增强市场竞争力，提高经济效益。紧跟东风公司结构调整步伐，推进汽车零部件产品结构调整。整合零部件资源，引导资源向优势企业集中，支持骨干企业与国内外优势企业合资重组。鼓励企业全方位开拓市场，不断扩大与东风公司各个板块的配套份额，争取与其他国内国际汽车知名企业建立配套关系，扩大社会销售量，提高市场占有率。

[FS:PAGE]

　　大力发展水电产业。加快汉江、堵河流域水能资源综合开发步伐，通过项目开发带动流域开发，以流域开发促进区域经济发展。抓好黄龙滩扩机、松树岭、鄂坪、三里坪等在建工程建设，继续抓好潘口、龙背湾、孤山等项目的前期工作，争取早日开工建设。加快电网建设步伐，建成竹山、丹江口、柏林220千伏变电站，启动实施竹溪输变电工程建设。

　　高起点发展医药化工产业。运用现代生物技术，深度开发黄姜、杜仲、黄连等药用资源，形成激素类、中成药类等医药化工产品系列。大力支持以清大药业、十堰麦克、康迪制药、武当药业为骨干的中药材加工企业，开发、引进中药新品种，带动全市医药产业快速发展。支持皂素加工企业联合重组，推广清洁化生产新技术、新工艺，延长产业链，带动黄姜产业持续健康发展。

　　加快发展绿色食品产业。深度开发魔芋系列保健食品和绞股蓝、猕猴桃等保健饮料产品，加快发展矿泉水、葡萄酒、系列黄酒等传统优势产品，工业化开发食用菌、干鲜野菜等土特产资源，在天然、保鲜、保健、无污染等方面做文章，形成规模，创出名牌。

　　（三）突破性发展第三产业，提升产业层次。

　　壮大旅游支柱产业。抢抓南水北调工程建设契机，高标准编制水体旅游规划，整合全市旅游资源，加快形成以武当山为中心，以山水相依、山城一体为特色的旅游格局。建立多元化旅游投资体系，引进实力雄厚的投资主体参与风景区开发。积极推进旅游重点项目建设，大力开发具有地方特色的旅游产品。加大旅游宣传促销力度，积极推销精品线路，提高旅游产品的市场竞争力。力争接待国内外游客增长10%，旅游总收入增长15%，旅游创汇增长20%以上。

　　加快构建现代流通体系。围绕优势资源和支柱产业，加快建设鄂西北小商品市场等特色市场，加强边贸市场和城镇集贸市场建设，形成联接城乡、辐射周边的商品市场体系。支持中国（十堰）汽配城扩大销售，增强市场竞争力和社会影响力。创新营销体系和方式，大力发展连锁经营和代理制。加快发展现代物流业，制定全市物流产业发展规划，引进国内外大型物流企业，培育建设物流园区，构筑辐射全市和周边的物流体系。

　　着力扶持新兴服务业。加强信息网络基础设施建设，整合信息资源，积极推进电子商务、电子政务和企业信息化建设。规范房地产市场，推进物业管理市场化，完善住房公积金制度，促进房地产业健康发展。支持餐饮、交通运输、社区服务等就业能力强的服务业发展，鼓励科技、教育、文化、体育、医疗保健、金融保险等产业发展，促进会计、律师、咨询、资产评估等中介服务业规范有序发展。培植汽车、住房等新的消费热点，推动消费结构升级，扩大消费需求。

　　（四）加强项目建设，培植发展后劲。

　　集中力量建设一批重点产业项目。全力支持东风公司的悍马越野车、客车、发动机、车身改造、变速箱等重大项目建设。加快推进万向通达和普林汽车零部件加工、奥斯达自动调整臂、万益复合扭震减震器、神河半挂集装箱运输车、农夫山泉新增生产线、郧县干法水泥等重点项目建设。

　　大力加强交通建设。全力支持十漫高速公路和襄渝铁路复线建设。推进干线路网建设，改造路面工程111公里，开工建设房县城关至温泉一级公路，基本完成将军河大桥主体工程，确保丹江口二桥竣工通车，力争高速公路客运站项目早日开工。继续加强农村公路建设，实现乡乡通油路目标。

　　加大城镇规划建设管理力度。严格实施省政府批复的《十堰市总体规划》，做好停车场、农贸市场、教育发展用地等项目的控制性详规。加快推进市区道路建设，支持县市城区道路、管网、绿化等各项基础设施建设。抓好市政设施维护管理，加强公共绿地建设和市容市貌管理。

　　全力服务南水北调中线工程建设。切实抓好移民搬迁安置、水源水质保护等工作，积极推进施工大桥建设，精心做好大坝加高工程的生产、生活服务保障工作。积极争取优惠政策、对口支援和补偿项目，力争丹江口库区小流域治理、水土保持、水资源保护、水污染治理等列入国家项目。

　　（五）加大改革开放力度，为经济发展注入新活力。

　　进一步深化各项改革。巩固农村税费改革成果，落实农业税免征政策，完成乡镇综合配套改革任务，加强涉农收费监管，坚决防止农民负担反弹。依法完善农村土地二轮延包。深化国有集体企业改革，完善国有资产监督管理体制，积极引导改制后企业健全现代企业制度，鼓励企业合资合作，实现资源优化配置。推进市政公用行业改革，鼓励社会资本参与市政公用设施建设，加快园林、环卫、供水等行业的市场化进程。大力发展民营经济，进一步落实优惠政策，营造公平竞争环境，不断提高民营经济的产业层次和企业素质。

　　努力提高对外开放水平。坚持外资内资并举、招商安商并重，进一步突出企业的招商主体地位，面向重点国家和地区组织好主题招商活动，大力推行园区整体招商、产业链招商、以商招商和小分队招商等形式，力争招商引资取得新突破。加强重点出口基地建设，培养和引进外经贸人才，培育和发展外经贸企业集团，加大国际市场开拓力度，不断扩大出口规模。支持十堰高新区和东城、西城等开发区突出产业特色，加快关键零部件产业基地、高新技术产业孵化中心和特色园区建设，更好地发挥对外开放的窗口作用和区域经济的龙头作用。

　　（六）做好财税金融工作，支持经济社会发展。

　　加强财税工作。继续推进财政管理体制改革，加强预算管理，强化国库集中支付，严格实行“收支两条线”，扩大政府采购范围和规模。综合运用财税政策和财政手段，支持基础财源、骨干财源、新兴财源项目建设。坚持依法治税，切实做到应收尽收，确保主体税种稳定增长，增加地方可用财力。调整优化支出结构，下大力降低行政运行成本。

　　完善金融服务。大力推进“信用十堰”建设，加强同金融部门的沟通与衔接，积极推荐优质项目，扩大信贷投资规模。建立以中小信用担保机构为主体、商业性担保机构为补充的担保体系，完善下岗失业人员小额担保贷款风险补偿机制，解决中小企业和民营经济担保难、贷款难问题。继续推广农村小额信贷，管好用好支农再贷款，积极支持农业和农村经济发展。

　　（七）切实关心群众利益，全力维护稳定大局。

　　千方百计扩大就业。落实就业再就业优惠政策，推进就业服务的制度化、专业化、社会化。继续抓好职业技能和创业技能培训，缓解就业结构矛盾。大力发展劳动密集型产业、非公有制经济和中小企业，积极开发就业岗位，加强就业指导，着力解决好困难行业、困难群体的就业再就业问题。加大劳动保障执法监察和劳动争议仲裁工作力度，维护劳动者合法权益。

　　提高社会保障能力。以民营企业从业人员和灵活就业人员为重点，进一步加大社会保险扩面征缴力度，增强基金支撑能力。规范社会保险基金的使用和管理，确保基金运营安全。继续巩固“两个确保”成果，实现“两金一费”按时足额发放。完善城市居民最低生活保障制度，提高最低月补助标准。切实解决失地农民和受灾群众的生产生活困难，保障弱势群体合法权益，加强“福星工程”建设，支持残疾人事业发展。

　　高度重视人民群众生产生活安全。落实安全生产责任制和责任追究制，继续抓好交通安全、矿山安全、消防安全和烟花爆竹等专项整治，坚决遏制重特大事故的发生。深入实施放心食品、药品工程，依法严厉打击食品、药品违法犯罪活动，确保人民生活安全。

　　全力维护社会稳定。加强社会治安综合治理，完善社会治安防控体系，依法打击各种刑事犯罪活动和“法轮功”等邪教组织活动，坚决扫除“黄、赌、毒”等社会丑恶现象。认真做好群众来信来访工作，坚持依法办事，维护群众合法权益，完善矛盾纠纷调处工作机制，把各类社会矛盾化解在萌芽状态，积极预防、妥善处置突发性和群体性事件。

[FS:PAGE]

　　（八）全面发展社会事业，努力构建和谐十堰。

　　大力推进科技进步和教育发展。引导和支持企业成为技术创新的主体，加大产学研合作力度，提高科技创新能力。大力推进高新技术产业发展，组织实施汽车关键零部件、南水北调中线工程水源区可持续发展、生物技术与新医药、有机生态农业及农业产业化等四个科技专项。积极做好科学普及工作，推进区域性科普展教中心建设。巩固“普九”成果，向普及高中阶段教育迈进。推进教育资源整合，优化城乡学校布局，扩大城区中小学办学规模，改善农村住宿学校基础设施条件，大力发展高等教育和职业教育。鼓励发展民办教育，积极探索多种形式的办学路子。实施人才强市战略，完善人才培养、使用、引进、激励工作机制，有序推进以党政人才、企业经营管理人才和专业技术人才为主体的人才队伍建设。

　　加快发展文化体育、广播电视、医疗卫生事业。加强基层文化体育设施建设，广泛开展丰富多彩的群众文体活动。推进市豫剧团改革发展，打造精品剧目。做好世界文化遗产保护管理和库区文物抢救保护工作。实施广播电视光纤村村通、有线电视农村入网、广播电视数字化工程，提升广播电视事业发展水平。坚持“扫黄打非”，打击盗版侵权行为，净化网络市场和文化市场。全面实施公共卫生体系建设规划，提高公共卫生突发事件应急处理能力和重大传染性疾病防治能力。加强第二轮农村初保工作，继续抓好农村合作医疗试点，提高农村医疗卫生服务水平。

　　实施可持续发展战略。加强计划生育依法管理和优质服务工作，推进计生服务站国债项目建设，继续执行“一票否决制”，坚持稳定低生育水平，不断提高出生人口素质。严肃查处利用非法手段进行胎儿性别鉴定和选择性别终止妊娠行为，遏制出生人口性别比升高趋势。大力发展循环经济，走节约型发展道路。加强资源保护和合理开发，依法查处非法采矿、乱采滥挖等破坏和浪费矿产资源的行为。加大生态建设力度，大力开发生态产业。继续推进退耕还林、天然林保护等国家重点生态工程，完成造林40万亩。加强丹江口湿地、赛武当、十八里长峡、堵河源等自然保护区建设。加大地质灾害防治力度。

　　深入开展精神文明创建活动。加强社会公德、职业道德和家庭美德教育，特别要加强对青少年的思想道德教育。以社区创建为重点，全力推进文明城市创建活动。以“六大工程”为载体，进一步深化农村精神文明建设。加大“窗口行业”的创建力度，提高服务质量。积极推进非公有制企业文明创建活动，扩大精神文明建设的覆盖面。抓好316国道省级文明路创建工作。巩固提高省级卫生城市成果，争创国家级卫生城市。深入开展全民国防教育，巩固军政军民团结，加强民兵预备役建设，争创双拥模范城五连冠。

　　（九）加强政府自身建设,提高依法管理经济社会事务的能力。

　　进一步转变政府职能。在继续加强经济调节和市场监管职能的同时，更加重视政府的社会管理和公共服务职能，把精力更多地放到推进社会发展和解决民生问题上来，不断提高驾驭社会主义市场经济的能力、发展社会主义民主政治的能力、建设社会主义先进文化的能力、构建社会主义和谐社会的能力。深化行政管理体制改革，尽快把新一轮机构改革推进到位。进一步减少审批事项，提高办事效率，优化投资环境。加强政风建设，强化群众观念，完善信访接待日制度、市长电话制度；增强服务意识，完善行政服务中心职能，提高服务质量；推行政务公开，继续开展民主评议行风、企业评议政府部门、社会公众评议政府机关等活动。

　　大力推进民主法制建设。严格按照法定的权限和程序履行职责，认真执行人大及其常委会的决定决议，自觉接受人大监督，接受政协民主监督，主动加强同民主党派、工商联和社会各界的联系，建立和完善重大问题集体决策制度、专家咨询制度、社会公示制度、社会听证制度和决策责任制度，推进科学民主决策。加强基层政权建设，认真做好乡镇换届选举工作。全面贯彻《依法行政实施纲要》，提高依法行政水平。认真做好全市“四五”普法总结验收工作。

　　切实加强廉政建设。抓好责任分解、责任考核、责任追究三个环节，把廉政建设责任制落到实处，加强对政府机关工作人员的严格要求、严格教育、严格管理、严格监督。坚持标本兼治、综合治理，加大源头治腐力度，严格执行建设工程招投标、经营性用地使用权挂牌出让、产权公开交易等制度。加强对征地和拆迁补偿资金使用的监管，解决企业重组改制中损害职工合法权益的问题，深入治理教育乱收费，纠正医药购销和医疗服务中的不正之风。

　　今年我们还要做好“十一五”规划和2020年远景目标的制定工作，这是新一届政府的一项重要工作，也是全市人民普遍关注的一件大事。要深入调查研究，积极问策纳言，着重做好汽车、三农、教育、科技、就业和社保等专项规划，加强部门规划、项目规划的制定工作，确保高质量、高标准完成规划编制任务，为十堰未来发展绘制既切实可行又鼓舞人心的宏伟蓝图。

　　各位代表，努力为人民群众办实事，是各级政府应尽的职责。这里，我代表市人民政府郑重承诺，今年要为全市人民群众着力办好十件实事：一是实现城镇新增就业岗位3万个，安置就业3万人。二是筹措资金1400万元，完成1万贫困人口、500户受地质灾害严重威胁居民的搬迁任务。三是筹措资金300万元，为1.5万名农村贫困中小学生免费发放教科书和减免杂费。四是继续实施“福星工程”，基本实现愿意集中供养的五保对象集中供养。五是在国家免征贫困县市农业税的基础上，市财政自筹资金100万元，用于免征茅箭区、张湾区、十堰高新区农民的农业税。六是筹措资金600万元，基本解决全市贫困人口住茅草屋问题。七是筹措资金1.5亿元，修建通乡油路280公里、通村油路500公里，实现“乡乡通油路”目标。八是筹措资金2000万元，新建沼气池1.5万口，解决2万人饮水安全、5.5万人饮水困难。九是筹措资金5.5亿元，加快推进天津路、甘肃路、重庆路、浙江路等城区道路的建设。十是加快推进市博物馆、游泳馆及百二河健身长廊工程建设。

　　各位代表！过去五年的成就，奠定了我市经济社会发展的坚实基础；未来发展的机遇，必将坚定全市人民奋发有为的信心。我们相信，新一届政府一定会在市委的正确领导下，团结依靠全市人民，与时俱进，锐意进取，励精图治，扎实工作，以更加辉煌的业绩，创造十堰更加美好的未来。
